# Supplementary material for: Record statistics of bursts signals the onset of acceleration towards failure
Source: Sci Rep. 2020 Feb 13;10:2508. doi: 10.1038/s41598-020-59333-4 (PMC7018714; doi:10.1038/s41598-020-59333-4)
Supplement: Supplementary file 1 — Supplementary Information. [file 41598_2020_59333_MOESM1_ESM.pdf]

Supplementary information for

V. Kádár[1], G. Pál[1,2], and F. Kun[1,2*]

Record statistics of bursts signals the onset of acceleration towards failure

[1]Department of Theoretical Physics, Doctoral School of Physics, Faculty of Science and Technology, University of Debrecen, H-4010 Debrecen, P.O.Box: 5, Hungary
*Corresponding author Email: ferenc.kun@science.unideb.hu

[2]Institute of Nuclear Research (Atomki), H-4026 Debrecen, Poroszlay út 6/c, Hungary

SI1: Derivation of the brittle - quasi-brittle phase boundary

The probability density $p(\varepsilon_{th})$ of the breaking thresholds of fibers has a power law functional form

$$p(\varepsilon_{th}) = D\varepsilon_{th}^{-(1+\mu)}, \tag{S1}$$

over the range $\varepsilon_{min} \leq \varepsilon_{th} \leq \varepsilon_{max}$. The cumulative distribution of thresholds reads as

$$P(\varepsilon_{th}) = \frac{\varepsilon_{th}^{-\mu} - \varepsilon_{min}^{-\mu}}{\varepsilon_{max}^{-\mu} - \varepsilon_{min}^{-\mu}}. \tag{S2}$$

Since equal load sharing is assumed after fiber breakings, the constitutive equation $\sigma(\varepsilon)$ of the bundle can be obtained from the general form $\sigma(\varepsilon) = E\varepsilon[1 - P(\varepsilon)]$ by substituting the distribution function $P(x)$ from Eq. (S2) [P1,P2]

$$\sigma(\varepsilon) = \begin{cases} \varepsilon, & 0 \leq \varepsilon \leq \varepsilon_{min}, \\ \dfrac{\varepsilon(\varepsilon^{-\mu} - \varepsilon_{max}^{-\mu})}{\varepsilon_{min}^{-\mu} - \varepsilon_{max}^{-\mu}}, & \varepsilon_{min} \leq \varepsilon \leq \varepsilon_{max}, \\ 0, & \varepsilon_{max} < \varepsilon. \end{cases} \tag{S3}$$

For finite cutoff strength $\varepsilon_{max} < +\infty$ the constitutive curve $\sigma(\varepsilon)$ has a maximum whose position ε_c and value σ_c define the fracture strength of the

bundle. The critical strain and stress depend on the disorder parameters of the model

$$\varepsilon_c = \varepsilon_{max}(1-\mu)^{1/\mu}, \tag{S4}$$

and

$$\sigma_c = \frac{\mu(1-\mu)^{1/\mu-1}\varepsilon_{max}^{1-\mu}}{\varepsilon_{min}^{-\mu}-\varepsilon_{max}^{-\mu}}. \tag{S5}$$

The bundle exhibits a perfectly brittle behaviour if the breaking of the weakest fiber triggers immediate abrupt failure. This occurs when the critical strain ε_c coincides with the lowest breaking threshold ε_{min}. It follows that at any μ exponent there exists a critical upper bound

$$\varepsilon_{max}^{c} = \frac{\varepsilon_{min}}{(1-\mu)^{1/\mu}}. \tag{S6}$$

below which the bundle fails abruptly. The above equation defines the phase boundary between the brittle and quasi-brittle phases of the model in Fig. 1 of the manuscript.

SI2: Derivation of the average number of breaking fibers triggered by the breaking of a single fiber at a strain ε

The load $\sigma = E\varepsilon$ dropped by the broken fiber is equally shared by the intact ones of number $N[1-P(\sigma)]$, giving rise to the stress increment $\Delta\sigma = \sigma/N[1-P(\sigma)]$. Multiplying $\Delta\sigma$ with the probability density $p(E\varepsilon)$ of failure thresholds and with the total number of fibers N the average number of triggered breakings a can be cast into the form

$$a(\varepsilon) = \frac{E\varepsilon p(E\varepsilon)}{1-P(E\varepsilon)} = \frac{\mu}{1-\left(\dfrac{\varepsilon}{\varepsilon_{max}}\right)^{\mu}}, \tag{S7}$$

which is Eq. (2) of the manuscript. Note that the Young modulus is $E=1$.

SI3: Supplementary figure to illustrate the behaviour of the average value of the largest record size and lifetime as function of the distance from the critical point of brittle failure

In the ductile phase of the system $\varepsilon_{max} = +\infty$, the record breaking process accelerates as the disorder exponent approaches the critical point of brittle failure $\mu \to \mu_c(\varepsilon_{max} = +\infty) = 1$. To quantify this behaviour we determined the average value of the largest record size $\langle \Delta_r^{max} \rangle$ and of largest

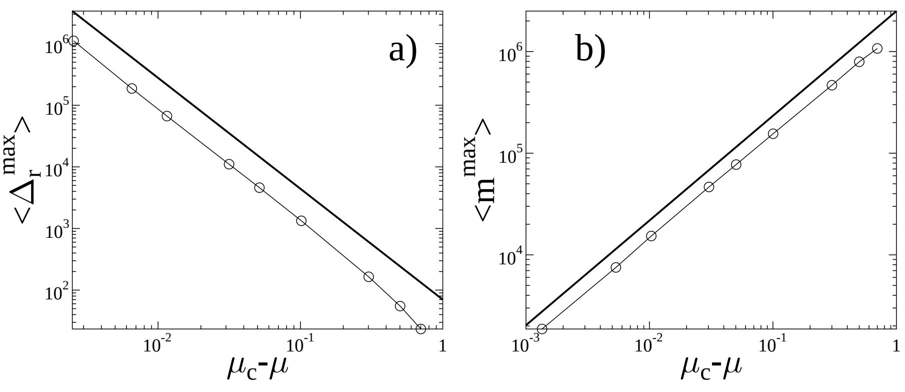

Figure S1: The average value of the largest record size (a) and largest record lifetime (b) as function of the distance from the critical point $1 - \mu_c$ on double logarithmic scale. The straight lines represent power laws of exponent $\alpha = 1.8$ (a) and $\beta = 1.0$ (b).

waiting time $\langle m^{max} \rangle$ that occurred up to failure as function of μ. Figure S1 presents the two quantities as function of the distance from the critical point $\mu_c - \mu$ with $\mu_c = 1$. Both curves can be very well described by straight lines, which implies the power law functional forms

$$\langle \Delta_r^{max} \rangle \sim (\mu_c - \mu)^{-\alpha}, \qquad \langle m^{max} \rangle \sim (\mu_c - \mu)^{\beta}, \tag{S8}$$

with the critical exponents $\alpha = 1.8(5)$ and $\beta = 1.0(5)$.

SI4: Supplementary figure of the average size of records $\left\langle \Delta_r^k \right\rangle$ at finite cutoff strength

Figure S2 illustrates that in the quasi-brittle phase of the system, when the cutoff strength of fibers is finite, the average size of records monotonically increases with the record rank similarly to the ductile phase.

References

[P1] Zs. Danku and F. Kun, *Fracture process of a fiber bundle with strong disorder*, Journal of Statistical Mechanics: Theory and Experiment **2016**, 073211 (2016).

[P2] V. Kadar, Zs. Danku, F. Kun, *Size scaling of failure strength with fat-tailed disorder in a fiber bundle model*, Physical Review E **96**, 033001

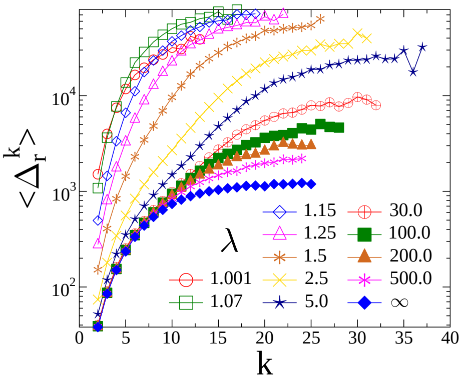

Figure S2: Average size of records $\left\langle \Delta_r^k \right\rangle$ as a function of the record rank k for several values of the cutoff strength of fibers λ at the disorder exponent $\mu = 0.7$.

(2017).
